# Supplementary material for: Molecular Diagnosis in Hymenoptera Allergy: Comparison of Euroline DPA-Dx and ImmunoCAP
Source: Toxins (Basel). 2025 Jun 19;17(6):310. doi: 10.3390/toxins17060310 (PMC12197634; doi:10.3390/toxins17060310)
Supplement: Supplementary file 1 [file toxins-17-00310-s001.zip › toxins-3524921-supplementary.pdf]

Supplementary Materials: Molecular Diagnosis in Hymenoptera Allergy: Comparison of Euroline DPA-Dx and ImmunoCAP

Lluís Marquès, Arantza Vega, Federico de la Roca, Carmen Domínguez, Víctor Soriano-Gomis, Teresa Alfaya, Laia Ferré-Ybarz, José-María Vega, Mario Tubella and Berta Ruiz-León

Table S1. Frequency of positive (> 0.35 kU/L) results to the different components for each technique. ImmunoCAP® and Euroline® class score (average with S.D.) Comparison with Spearman's correlation. All the patients tested with ImmunoCAP® were also tested with Euroline®. Some cases tested with Euroline® were not tested with all allergens of ImmunoCAP®. The Vs and Pd venoms in ImmunoCAP® are enriched with antigen 5 [10]. Am: Apis mellifera, Vs: Vespula spp, Pd: Polistes dominula.

Allergen	Frequency of positive result n (%)		Average class score (S.D.)		Spearman correlation coefficient (p-value)
	ImmunoCAP®	Euroline®	ImmunoCAP®	Euroline®	
**Am**	64 (45.1) (n=142)	69 (46) (n=150)	1.26 (1.8)	1.43 (2.03)	0.733 (<0.001)
**Api m 1**	32 (26.7) (n=120)	26 (17.2) (n=151)	0.71 (1.4)	0.47 (1.04)	0.726 (<0.001)
**Api m 2**	11 (12.4) (n=89)	29 (19.2) (n=151)	0.28 (0.78)	0.72 (1.48)	0.761 (<0.001)
**Api m 10**	31 (33.7) (n=92)	38 (25.3) (n=151)	0.9 (1.5)	1.39 (2.02)	0.949 (<0.001)
**Vs**	104 (69.3) (n=150)	121 (80.1) (n=151)	1.71 (1.44)	1.83 (1.44)	0.697 (<0.001)
**Ves v 1**	63 (50.8) (n=124)	50 (33.3) (n=151)	1.08 (1.35)	0.7 (1.25)	0.46 (<0.001)
**Ves v 5**	63 (45) (n=140)	94 (62.3) (n=151)	1.01 (1.33)	1.96 (1.97)	0.852 (<0.001)
**Pd**	115 (77.7) (n=148)	54 (35.8) (n=151)	1.97 (1.48)	0.62 (1.01)	0.588 (<0.001)
**Pol d 5**	66 (47.5) (n=139)	79 (52.3) (n=151)	1.13 (1.4)	1.51 (1.82)	0.778 (<0.001)
